# Editorial: Men who experience interpersonal violence: perspectives from research to intervention

**DOI:** 10.3389/fpsyg.2023.1247174

**Published:** 2023-07-21

**Authors:** Keren Gueta, Anat Ben-Porat

**Affiliations:** ^1^Department of Criminology, Bar-Ilan University, Ramat-Gan, Israel; ^2^The School of Social Work, Bar-Ilan University, Ramat-Gan, Israel

**Keywords:** sexual abuse, intimate partner violence, masculinities, victimization, gender

Interpersonal violence, including sexual assault and intimate partner violence (IPV), is a global and cross-cultural phenomenon. Over the past 50 years, academics and practitioners have strived to comprehend the phenomenon better and find practical solutions. Scholars who theorized about IPV several decades ago (e.g., Dobash and Dobash, 1978) focused on male-perpetrated violence toward female partners, using terms such as “wife abuse” and “violence against women.” As such, the early research on IPV was geared toward men's violence, while the victimization of men and boys was essentially ignored. Today, new avenues for developing this topic have been created by research and a theoretical understanding of contemporary masculinities, rarely included in studies of male interpersonal violence in the past.

Given the paucity of research worldwide, by looking at the current Research Topic we have aimed to shed light on this population of men as victims of interpersonal violence, emphasizing the unique characteristics associated with gender and male identity that shape their experience as victims.

Interpersonal violence against men has been linked with a variety of negative outcomes. Such outcomes have been evidenced on the physical front (e.g., cardiovascular disease), the mental/emotional front (e.g., depression, suicidal ideation), and the social front (e.g., isolation). To gain an understanding of male victims of interpersonal violence, it is crucial to take into consideration traditional masculine constructs such as machismo. Men may have gender-specific internal and external impediments to seeking help. For example, as a result of hegemonic masculinity, they may have difficulty merely asking for help, or they may fear humiliation or a lack of support when they do ask for help. Thus, we must develop a solid conceptual understanding of men's victimization experiences that would be useful for both clinicians and policymakers.

The Research Topic consists of five original articles focusing on various aspects of male victimization. First, there is the developmental perspective on the origin of male victimization throughout childhood, adolescence, and adulthood. In addition, the Issue includes multiple arenas and contexts in which boys and men may be particularly vulnerable to and victims of interpersonal violence, such as the workplace or online. Furthermore, this Research Topic addresses the paucity of research about the overlap between victimization and perpetration. This gap in the literature may stem from the way victimization and perpetration are viewed—that is, as being at the opposite ends of two poles, rather than actually sharing certain aspects.

Leiding et al. study cuncludet that violence is a recognized risk factor for health problems, and that men who are both victims and perpetrators of violence make up the largest group of men on the violence continuum. They also have the worst health outcomes, because of the severe and wide-ranging violence they experience, and therefore heightened vulnerability. To prevent violence in the future, and to help both victims and perpetrators, intervention and prevention programs must be developed. Doing so will help these men first to identify and then to deal with maladptive behaviors. In addition, healthcare professionals must take into account, in a comprehensive manner, potential health-related outcomes when treating individuals who are exposed to violent activities.

Ngubane et al. explored the experiences of male perpetrators of rape in South Africa who were in jail. The authors aimed to identify both the social context and the individual childhood experiences that might have contributed to the men's perpetration of rape. The findings provide insights into the complicated dynamics of the abuse cycle and the way in which criminal behavior may evolve, with individuals beginning as victims and ending up as perpetrators. The study makes clear that interventions are needed to reduce childhood trauma exposure and improve the contexts (both personal and social) in which those at risk for childhood neglect and abuse live.

Wu et al. paper indicate among high school students the positive correlation between mobile phone addiction and depression. Cyberbullying victimization and perpetration were found to mediate this relationship, with boys who were victims of cyberbullying being more likely than girls to become perpetrators of cyberbullying. The authors suggest that forceful response measures must be taken in this context, especially for boys who have an addiction to their mobile phones. In addition, these boys must be provided with help in coping with cyberbullying crises and with their emotions.

The paper by Chen et al. contributes to understanding how prevalent psychological abuse is among Chinese adolescents as well as the negative impact of this kind of abuse on self-esteem and social support. In addition it highlight that self-esteem of psychological abuse survivors must be improved so that the negative effect of low self-esteem on social support will be reduced. To lessen the prevalence of psychological abuse, the authors suggest that parents be given information about positive parenting strategies by governments and communities. In terms specifically of the gender aspect of psychological abuse among Chinese adolescents, male adolescents were found to be more likely to experience psychological abuse than female adolescents. This finding aligns with findings from previous studies. Different parenting behaviors, stemming in part from stricter rules for the expression of emotions among boys/men in traditional Chinese culture, may account for this finding.

Finally, Zedlacher and Yanagida examined whether female perpetrators received more moral anger and blame from observers than men in ambiguous psychological workplace mistreatment. The findings showed that female perpetrators received harsher judgments than did men in the context of the target being female, and when the mistreatment was not taken into account. Female targets received less blame when the perpetrator was female (i.e., rather than male). Attributional biases did not come into play with regard to male targets (as opposed to female targets). The study findings suggest that target gender and type of mistreatment largely determine gender biases in perpetrator-blaming.

## Author contributions

All authors listed have made a substantial, direct, and intellectual contribution to the work and approved it for publication.
